# LOSARTAN REJUVENATES SERUM AND HEART METABOLOME OF AGED MICE BY ANGIOTENSIN DEPENDENT AND INDEPENDENT MECHANISMS

**DOI:** 10.1093/geroni/igae098.3668

**Published:** 2024-12-31

**Authors:** Michael Bene, Ruth Marx, Laura Powell, Luigi Ferrucci, Mohammed Khadeer, Ruin Moaddel, Michelle Rudek, Jeremy Walston

**Affiliations:** Johns Hopkins School of Medicine, Baltimore, Maryland, United States; Johns Hopkins University, Baltimore, Maryland, United States; Johns Hopkins University, Baltimore, Maryland, United States; National Institute on Aging, Baltimore, Maryland, United States; National Institute on Aging, Baltimore, Maryland, United States; National Institute on Aging, Baltimore, Maryland, United States; Johns Hopkins University, Baltimore, Maryland, United States; Johns Hopkins University, Baltimore, Maryland, United States

## Abstract

Emerging evidence suggests losartan, a selective angiotensin II receptor type 1 (AT1) antagonist, may reduce frailty risk and improve physical function in older adults. Losartan is metabolized to EXP3174 (a potent AT1 antagonist) and EXP3179 (a PPARγ agonist). To elucidate mechanisms through which losartan may reduce frailty and improve physical function we performed targeted metabolomics in serum and heart samples from young (4 months) and aged (24-28 months) mice. Aged mice treated with losartan displayed a global shift towards a more “youthful” serum metabolome (ρ=-0.22, p=0.01) and a stronger “youthful” shift in the heart metabolome (ρ= -0.49, p =3.6e-8), with 71/120 and 74/115 measured metabolites shifting in abundance opposite to age respectively. Serum of AT1KO mice showed no significant metabolomic change but displayed a similar “youthful” shift when treated with losartan, indicating losartan’s effect is AT1 independent. Supporting this, a mediation analysis of serum levels of losartan and EXP3174 revealed serum losartan had a significant correlation to the metabolomic aging signature that was not mediated by EXP3174. In contrast to serum, AT1KO alone shifted the heart metabolome to a more ‘youthful” state suggesting losartan’s effect on the heart may be partially AT1 dependent. Interestingly, AT2KO mice also displayed more “youthful” serum and heart metabolomes, but when treated with losartan produced an interference effect blocking the “youthful” shift seen with either AT2KO or losartan alone. Losartan’s “rejuvenating” effect on serum and heart metabolome was associated with improved survival in WT mice (log-rank, p =0.015) further supporting improvement in aging trajectory.

